# The future of human cerebral cartography: a novel approach

**DOI:** 10.1098/rstb.2014.0171

**Published:** 2015-05-19

**Authors:** Richard Frackowiak, Henry Markram

**Affiliations:** 1 The Human Brain Project, Centre Hospitalier Universitaire Vaudois, University of Lausanne, Lausanne 1011, Switzerland; 2 The Human Brain Project, Ecole Polytechnique Fedérale de Lausanne, Lausanne 1015, Switzerland

**Keywords:** cartography, map, brain, medicine, multi-scale

## Abstract

Cerebral cartography can be understood in a limited, static, neuroanatomical sense. Temporal information from electrical recordings contributes information on regional interactions adding a functional dimension. Selective tagging and imaging of molecules adds biochemical contributions. Cartographic detail can also be correlated with normal or abnormal psychological or behavioural data. Modern cerebral cartography is assimilating all these elements. Cartographers continue to collect ever more precise data in the hope that general principles of organization will emerge. However, even detailed cartographic data cannot generate knowledge without a multi-scale framework making it possible to relate individual observations and discoveries. We propose that, in the next quarter century, advances in cartography will result in progressively more accurate drafts of a data-led, multi-scale model of human brain structure and function. These blueprints will result from analysis of large volumes of neuroscientific and clinical data, by a process of reconstruction, modelling and simulation. This strategy will capitalize on remarkable recent developments in informatics and computer science and on the existence of much existing, addressable data and prior, though fragmented, knowledge. The models will instantiate principles that govern how the brain is organized at different levels and how different spatio-temporal scales relate to each other in an organ-centred context.

## 1. Introduction

**Figure 1.**
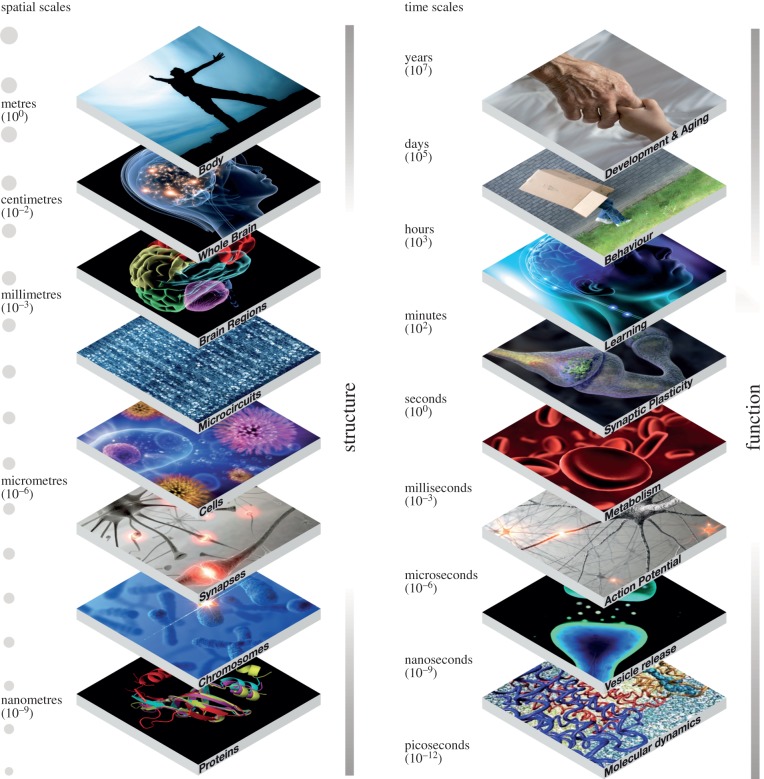
The brain is an intrinsically multi-scale, multi-level organ operating across spatial scales ranging from nanometres (proteins) to metres (the human body) and temporal scales from picoseconds (atomic interactions) to years (the lifespan of a human being).

‘An image is worth a thousand words'*.* Cerebral cartography in the modern sense means much more than anatomy (maps) or cerebral connections (routes). Rather, the aim is to generate atlases that use anatomical frameworks to organize and convey spatially and temporally distributed functional information about the brain at all organizational levels, from genes to cognition, and at all the relevant spatial and temporal scales. The ultimate brain atlas will, therefore, be an instantiation of a comprehensive multi-scale understanding of the brain. In short, a description of structural and functional principles at each scale and across all scales (figure 1), all leading to the plethora of manifestations of whole brain function, normal and abnormal.

Thirty years ago, most sciences were craft industries. Although high-energy physics and astronomy had big teams and expensive instruments—particle accelerators, telescopes—most published research came from highly skilled scientists, working in small laboratories, mostly in the wealthier nations. Neuroscience, basic and clinical, followed the same pattern. Focus was an essential element for success with grant applications. Even today, the world's 100 000 or so neuroscientists work essentially on their own, each developing his or her interest, each using established or innovative methods to dig deeper into one particular corner of the brain. Collaborative networks and consortia are forming, often as a result of changes in scientific funding policy, but culturally, even these new groupings remain in the world of the traditional hypothesis-led paradigm. The results have been astounding in scope, quality, amount and breadth. All our basic concepts have come from specialized investigations by scientists working within this traditional model. But today, that model faces serious challenges.

## 2. Historical considerations

After the pioneering work of Brodmann [1] and many others who followed him [2,3], a key development in human brain mapping was the introduction of maps using stereotactic coordinates to identify brain regions in three-dimensional space. This third dimension provided a vital tool for brain surgery in humans and experimental animals. In 1967, Jean Talairach and Pierre Tournoux published the first edition of their atlas of the human brain, which became a basic reference for the anatomical identification of brain areas localized in human functional brain imaging studies with positron emission tomography (PET) [4,5]. In 1982, George Paxinos published his famous atlas of ‘The Rat Brain in Stereotaxic Coordinates', which provided an equivalent framework for the rat [6], later going on to publish similar atlases for the rhesus monkey [7] and the mouse [8]. These works, published in print format, met a fundamental need. However, as time moved on, new exigencies resulted in a series of further developments.

Long before Talairach and Paxinos, Brodmann and others' work had been criticized for its reliance on subjective classifica tion criteria, poor reproducibility and an inability to account for inter-individual variation [9–11]. This criticism implied a need, on the one hand for objective methods of parcellation and on the other for strategies that capture anatomical variations between individual brains.

The former need was met by the introduction of new imaging technologies and methods for the measurement of various brain characteristics: computerized tomography (CT), magnetic resonance imaging (MRI: structural—sMRI; functional—fMRI; resting state—rsMRI; and diffusion weighted imaging—DWI; various forms of tractography and others). The availability of these new techniques led to a massive reduction of the effort needed to produce brain atlases.

At the same time, it became imperative to create standard schemata into which individual brains could be morphed so that images from a variety of individuals could be averaged, contrasted and analysed in various ways. This revolution turned human brain imaging from a radiological technique for medical use into a quantitative science. It also led to significant improvements in the accuracy, reliability and objectivity of imaging data and new knowledge about human brain regions. The result was an explosion in scientific productivity.

In parallel with such efforts, and especially after the introduction of MRI, researchers addressed the issue of inter-individual variability, developing probabilistic, population-based, analytical methods that combined computational techniques and database technology to map individual brains onto standard anatomical templates with spatial coordinates [12–14]. The new techniques, based on anatomical normalization into a standard brain space and novel statistical techniques designed to deal with multiple non-independent comparisons at a voxel level (e.g. as instantiated in the Statistical Parametric Mapping package [15]), made it possible to discover features and correlations impossible to detect in individual brains, and to atlas the brains of specific subpopulations (including subjects diagnosed with specific neurological or psychiatric diseases). Examples of this approach include the Montreal Neurological Institute's reference brain, averaged from 301 normal structural MRI scans, which became a *de facto* MRI standard in the human brain imaging community and the ‘Probabilistic atlas and reference system for the human brain’ produced by the International Consortium for Brain Mapping (ICBM) in 2001 [16]. In 1995, the Harvard Whole Brain Atlas [17,18] was launched, which provided a reference three-dimensional MRI/PET atlas of the human brain and which offered the community specific databases that combined imaging and other data relevant for collaborative analyses. Other examples of similar approaches include the Alzheimer's disease neuroimaging initiative (ADNI), an Internet-accessible database that contains MRI, PET and other data related to ageing and Alzheimer's disease [19,20], the NIMH Paediatric Imaging Study [21,22] and the Finnish Twin Registry [23].

Thanks to the availability of new imaging methods and the rapidly increasing power and falling costs of computer memory and database technology, many of these studies were able to explore aspects of the brain that had not been mapped previously. Thus, the Harvard Whole Brain Atlas provides detailed brain maps not only for the normal brain but also for brains of patients with cerebrovascular, neoplastic, degenerative, inflammatory and infectious diseases. Similarly, the ICBM has deliberately designed its atlas to quantify variance in the human brain as a function of time (the atlas contains data from 7000 subjects between the ages of 18 and 90). It also includes demographic, behavioural, clinical and genotype data so permitting correlational and other analyses. The Human Connectome Project, launched in 2005, uses DWI to track white matter fibres, rsMRI to document functional connectivity, fMRI to explore effective connectivity, sMRI for spatial localization and separate or combined magneto- and electro-encephalography (MEG/EEG) for characterization of brain dynamics. A further enhancement was the combination of imaging with extensive genetic data to characterize brain connectivity and its variability in healthy humans.

Non-human studies have seen similar and parallel developments. Advanced database and Web technology have made it possible to collect and organize data generated using methods not applicable in humans, such as retrograde and anterograde tract tracing. To cite just one example, 1996 saw the launch of CoCo-Mac—a large electronic repository currently containing more than 40 000 experimental findings on anatomical connections in the macaque brain [24].

Gene sequencing and other -omic technologies [25–28], introduced during the late 1990s and in the first decade of the twenty-first century, together with large-scale sequencing and annotation efforts in humans, mice, chimpanzees and other species [29–33], have made it possible to expand the range of brain atlases to include more basic levels of brain organization. This effort, pioneered by the Allen Institute for Brain Sciences, has used *in situ* hybridization and micro-optical tomography to produce the first genome-wide atlas of mRNA expression in the mouse brain (*The Allen Mouse Brain Atlas*) [34,35]. This was soon followed by the *Allen Developing Mouse Brain Atlas*, which represents the development of mouse brain across four prenatal and three early postnatal time points [36], and the *Allen Mouse Diversity Study*, which characterizes seven strains of male and female C57BL/6J mice in three stages of oestrus. Similar techniques have been used to produce the *Allen Human Brain Atlas* [37]. In parallel with this work, the Allen Institute has combined tract tracing with (EGFP)-expressing adeno-associated viral vectors and high-throughput, serial, two-photon tomography to create the *Allen Mouse Brain Connectivity Atlas* and recently, to publish the first meso-scale representation of the mouse connectome [38].

Other organizations and research groups have exploited new technologies to provide brain maps for other species, to cover new levels of description and to offer maps with higher spatial resolution than was previously possible. For example, the National Institutes of Health-funded *BrainMaps* initiative offers brain atlas datasets for *Macacca mulatta*, *Chlorocebus aethiops*, *Felis silvestris catus*, *Mus musculus*, *Rattus norvegicus* and *Tyto alba* [39,40]. The *NIH Blueprint Non-Human Primate* (*NHP*) *Atlas* provides a suite of gene expression and neuroanatomical data with informatics tools for exploration of the cellular and molecular architecture of macaque brain at different stages of prenatal and postnatal development [41]. Forschungszentrum Jülich and the Montreal Neurological Institute's *Big Brain project* redefine traditional neuroanatomical maps, such as those of Brodmann and von Economo, providing the first ultrahigh-resolution, three-dimensional digital atlas of a human brain with a resolution of 20 μm [42]. The *BrainSpan Atlas of the Developing Human Brain*, created in a collaboration between the Allen Institute of Brain Sciences, Yale University and other institutions, provides a spatio-temporal assessment of microRNA expression throughout early human brain development [43]. The *Human Protein Atlas Project* has also produced relevant and useful data at the proteomic scale [44,45].

This brief historical review illustrates how advances in computer science, informatics, statistics and mathematics have helped industrialize the neuroscientific process and already impacted our understanding of the human brain. Although they were led by technology, these advances have entrained cultural changes in the way science is done. Collaboration, industrialization of data collection and open sharing of data are becoming commonplace in a way that parallels the evolution of other areas of science, notably in physics, astronomy, meteorology and the material sciences. Neuroscience, however, has been much slower in taking up the advantages provided by the informatics explosion of the last decade and a half.

## 3. Limitations of experimental mapping

Recent years have seen enormous progress. Remarkable functional data from opto-genetic techniques in animal models and clarification of post-mortem brains demonstrate that technology is continuing to advance: a recent Chinese study has achieved Golgi staining of a whole rodent brain in three dimensions at 1 μm resolution.

Nonetheless, much of brain space remains *terra incognita* and most mammalian species have yet to be investigated. Furthermore, the resolution and depth of many pioneering maps are still limited by technology. For example, the Allen Institute's *Human Brain Atlas* represents relatively large blocks of tissue, containing many thousands of neurons in a single volumetric representation on a 100–300 μm grid [34]. *The Human and Mouse Connectome* projects are currently restricted in their scope to the meso-scale [38,46,47]. *BigBrain* provides 20 μm resolution, a halfway house between the mesoscopic resolutions of human brain maps collected in life and equivalent microscopic studies [42].

Many have suggested that simple living organisms with primitive nervous systems (such as *Caenorhabditis elegans*) could give us vital clues. In practice, however, reconstructing the nervous system of *C. elegans* has proved to be even more difficult than reconstructing the mammalian brain: the neurons (dendrites, axons) and synapses are too small to make the required physiological, pharmacological and biophysical recordings! At the opposite end of the spectrum, Genome Wide Association Studies (GWAS) searching for genes associated with brain diseases have produced relatively few significant observations and much of the available information is hard to interpret. Thus, a single mutation such as that of the Huntingtin gene, which is responsible for the neurodegeneration that gives rise to Huntington's disease through an unknown mechanism, can present with a variety of neurological, psychological or behavioural symptoms. *Vice versa*, a syndrome such as spino-cerebellar ataxia (SCA) is currently associated with 24 different mutations that have no obvious structural or functional relationship to each other. Implicit linear hypotheses linking genes to behaviour are for the most part false, which in retrospect is not surprising, but was not sufficiently obvious until sometime after the human genome was decoded.

There are many other examples in neuroscience where cartographic knowledge has failed to provide insights into cardinal features of behaviour. An obvious example is the organization of the primary visual cortex. Our current understanding of visual cortex has evolved from a great deal of work in North America (e.g. the school of S. Kuffler [48]) and in Europe (e.g. [49]). A cardinal example among this work is Hubel & Wiesel's [50] original Nobel-prize-winning model of the primary visual cortex, based on remarkable electrophysiological recordings, that has evolved over time into today's pinwheel model, which itself is based on imaging of oxyhaemoglobin variation in response to specific visual stimulation. Yet, despite these aesthetically and scientifically pleasing results that took decades to be generated and interpreted, there are still many aspects of visual perception we do not understand. For instance, many years of intensive study with ever more exact experimental procedures and methods have not given us an understanding of visual binding, or even apparently simpler functions such as spatial invariance. We do not understand how multiple visual features are processed and then interact in the same, albeit large, cortical space using neurons that do not reveal characteristic specificities related to differential functions, at least as described by histological and microscopic features. We do not know what level of biological detail is required to fully explain many visual phenomena. How will we achieve the new insights needed? Can we achieve them by collecting more and more data at ever-higher resolutions? Will it be possible in the foreseeable future to map all relevant cells and connections under all possible contexts and thus provide a mechanistic explanation? We are explicit in answering this rhetorical question in the negative.

A complete multi-level map of an individual human brain, at the resolution required for mechanistic explanations—and for detailed modelling and simulation—will have to represent the morphologies, physiology, subcellular and molecular architecture of some 10^11^ neurons and a similar number of non-neuronal cells (oligodendrocytes, astrocytes, microglia, etc.). More dauntingly still, it will need to represent between 10^14^ and 10^15^ synapses, each a complex sub-micron molecular machine in its own right, as well as the modulation of neuronal, glial and synaptic activity by neurotransmitters, peptides, hormones and other molecules. Even if some of this complexity is removed by grouping genes, proteins, cells and synapses into types, the task remains formidable. It is possible today to begin reconstructing connectivity among cells from stacks of EM images, to identify the morphology of cells from Golgi stains of clarified post-mortem brain tissue and to characterize the transcriptome of single cells. However, such techniques cannot presently be applied on the scale of the whole brain, let alone the whole human brain. As if these considerations were not enough, further complexity is added by temporal variations in the structure and function of individual brains (owing to stage of development, state of oestrus, health, environmental conditions, etc.), variations between individuals (according to gender, experience, etc.) and variations between species, where assumptions of homology are becoming increasingly difficult to sustain.

Probing even deeper levels of brain organization, at the level of the single-cell transcriptome and proteome, is even more difficult. How does the expression of subsets of genes, estimated at about 30% of coding genes, result in the construction of different types of neuron? How is each of the approximately 11 000 different proteins produced addressed and sent to different parts of this array of neuronal types? How many molecular pathways regulate the protein–protein interactions that contribute to this molecular machinery? With approximately a billion protein molecules in a single neuron, the number of pathways is potentially immense. Is it possible even to imagine a complete map of the reaction kinetics governing such interactions?

We conclude that despite the advances of the last half-century and the extraordinary methodological developments briefly reviewed above, information and knowledge relevant to aspects of brain physiology and anatomy have yet to be integrated into a comprehensive multi-scale brain model. The reason is simple: no adequate and comprehensive repository of such data and knowledge exists. Even if technologies continue to improve exponentially, it seems very unlikely that it will be possible to map more than a tiny part of brain territory in this detail at any time in the foreseeable future.

The Blue Brain Project, a Swiss initiative to reconstruct neocortical microcircuitry, is based on the most comprehensive molecular, cellular and synaptic dataset presently available for any brain region. The dataset is the result for thousands of cellular level experiments, carried out in many laboratories over a period of 30 years. Yet, together the data amount to only a minute fraction of that needed to provide a truly complete brain map. We, therefore, suggest a quite different strategy to solve the problem of producing a blueprint for how the brain is organized at its various levels of description, from genes to cognition and behaviour.

We contend that a complete understanding of the brain cannot be achieved at any one level of brain organization, no matter how well it is understood at that level. What is required is an integrated *multi-level* understanding. Obtaining all the genes expressed in the brain, or in each individual neuron, could be extremely useful for a behavioural neuroscientist, for example, but even if such a description were possible, a major effort would still be required to link it to behaviour. Recording from all the neurons in the brain might also be extremely valuable, if it were possible. But, the underlying machinery that produces the spikes would have to be described before the spike patterns could be fully understood. Identification of all the synapses connecting the brain's constituent neurons would provide a wealth of new insights into cerebral network architecture and guide interpretation of activity patterns. But to understand the connectome itself, it would be necessary to relate it to molecular, electrical and pharmacological properties and principles, and to the vast repertoire of emergent behaviours. Understanding how language is generated at the cognitive level would be an enormous step for cognitive science, but cannot be complete without understanding the machinery that supports language, and so on. No one level suffices to provide a comprehensive understanding of the brain. The strategy of keeping calm and carrying on in the same exploratory, piece-meal, scientific paradigm we are used to will not deliver a fundamental understanding of brain function. Even today, no one mind is capable of ingesting and comprehending the existing neuroscientific literature, even on a single topic such as vision. Our conclusion is that a new approach is needed, and this is what we predict will be the most significant development in the next 25 years of brain cartography. A comprehensive understanding of the brain implies an integrated explanation of its different organizational levels from genes to behaviour, and also an explanation of the way the different levels cooperate with each other.

## 4. A new paradigm

Despite the growing number of neuroscientists and exponential growth in neuroscience publications, and despite worldwide spending of around 7 billion Euro per annum, 1 billion in Europe, the benefits to society from neuroscience have been somewhat disappointing, leading politicians and industry to question its value. Finding new diagnostic tools and new treatments for brain diseases for which we have little mechanistic or causative understanding, has become increasingly difficult. The number of new drugs coming to market is falling and several pharmaceutical companies have stopped investing in the area. Neuroscience has had little authoritative to say about reducing the risk of brain disease through nutrition, education or social changes.

Traditional models of scientific research incentivise scientists to go for ‘big discoveries' and immediately move on, producing results that are difficult to replicate and leaving the data they have generated to fade away. Many clinical science studies are underpowered. A huge number are dropped or side lined because they do not support mainstream opinion or yield negative results. Meanwhile, neuroscience forges ahead without a plan for the rapidly growing volumes of data it produces, and without a curation process to secure its value for the future. Medicine, locked in a symptom-based diagnostic paradigm, struggles to move forward without a biological classification scheme for brain diseases. Plausibly, these are some of the key factors that are slowing progress towards a fundamental understanding of the principles of brain organization and the way it breaks down in diseases, eroding the impact of the considerable experimental and methodological progress that has been made.

Neuroscience lags behind many other sciences that have already embraced the digital era to build solid and common foundations for collaboration in their discipline. If we are to understand the brain across all scales and levels of organization, we need strong foundations to work together. Fortunately, we see signs of new paradigms and change on the horizon.

## 5. Big science

Big science emerges when a discipline is faced with radical, paradigm-changing opportunities leading to transformation of approaches and culture. Like calculus in the seventeenth century, information and communications technology (ICT) represents just such a transformative opportunity. Thanks to ICT, CERN's Large Hadron Collider (LHC) brought together physicists from 35 countries to explore the basic building blocks of matter—leading to the discovery of the Higgs Boson [51]. The Human Genome Project [29,30] mapped the whole human genome—immediately giving birth to a myriad of other projects: the Chimpanzee Genome Project [31], the Bonobo Genome Project [52], the Rice Genome Project [53], the 1000 Genomes Project [54,55], the 1000 Plant Genomes Project [56] and so on. Launched in 2000, the Sloan Digital Sky Survey (SDSS) mapped every stellar object in 35% of the sky [57,58]. NASA built the NASA Centre for Climate Simulation for scientists to conduct digital experiments, exploring the dynamics of past climate change and future scenarios [59]. Today nearly 60% of articles in astrophysics are based on the analysis of existing data. In the absence of supercomputers to analyse massive volumes of observational data and to run complex simulations and nonlinear models, and without Internet-based long-distance collaboration between scientists across the world, this kind of big science would not have been possible.

Our reflections over a half-decade suggest that it is now possible to create a unifying framework for neuroscience where new discoveries about brain organization can be placed and understood. On the one hand, there is an enormous number of facts in the archives of scientific articles about normal and diseased brains, or rather, different aspects of their anatomical, functional and biochemical organization. On the other, the last two decades have seen an explosion in computer science and informatics that has transformed the lives of individuals, impacted society and is having an effect on the cognitive function of a new generation of scientists bought up in the informatics age.

As we have already remarked, this explosion seems to have left basic and clinical neuroscience largely untouched and it is not obvious why this should be so. Other sciences have embraced the computing power of supercomputers; the ability to curate, organize and analyse massive volumes of data and thus to simulate and predict events and phenomena. Simulation modelling is, in fact, but just one of the many aspects of applied mathematics that have benefited from exponential growth in computing power. Advanced statistics, notably machine and deep learning, nonlinear multi-variate analysis and complexity mathematics are other relevant examples. The armamentarium is large and sets the stage for a paradigm shift from a purely hypothesis lead, reductionist approach to neuroscience, to a data-led, hypothesis-generating strategy, based on predictive simulation modelling.

## 6. The Human Brain Project

In 2012, these reflections were instantiated in a detailed application for a major competitive research grant from the European Commission's ‘Digital Connect’ directorate. The Human Brain Project (HBP) is a non-competitive, multi-disciplinary collaboration of over 110 institutions and 181 principal investigators who are working together on a single mission with a detailed roadmap that describes the work needed and the milestones to be achieved. The strategic work plan, made possible by advanced computer science and informatics, focuses on three mutually reinforcing areas of research: ‘future neuroscience’, ‘future medicine’ and ‘future computing’. In the first stage, the mission is to build six open infrastructures providing services for neuroinformatics, brain simulation, medical informatics, high performance computing, neuromorphic computing and neurobotics. The platforms, linked by a single unifying portal, will be supported by an existing network of European supercomputers, enhanced to include a dedicated Exabyte (10^18^ cps) supercomputer around the turn of the decade.

The European Commission's HBP is turning the challenge of mapping the human brain on its head. What we are trying to discover are the basic rules, the biological principles, underlying the construction of the brain we observe and catalogue, as we observe them at different stages of development and in different contexts. We conceive these principles as fundamental rules that govern the formation of a human brain with a spectrum of variations that explain inter-individual variability. To find them, we need a new ICT infrastructure capable of absorbing and analysing large amounts of neuroscientific and clinical data to derive a preliminary set of rules that govern the construction of the anatomical and functional features of the human brain. Much of the needed information resides in literature repositories and databases around the world, which already store over 3 million published papers. Initially, the digital reconstructions that we build with these rules will be incomplete. But, their shortcomings will expose what is not known and what is not understood and will point to the data needed to improve them. Since the reconstructions are based on principles suggested by available data, they can be systematically challenged and revised as new information becomes available. Since the links between different levels of biological organization are currently unknown, there is no equivalent method for formally testing concepts. However, simulated reconstructions can iteratively test the performance of different sets of rules and principles, within or between levels of organization, in describing the biological reality underpinning function. Iteration leads to adaptation and refinement of the rules identified initially and relates them to the rest of the architecture of the brain.

It is worth doing a mind-experiment to ask what principles and data would be needed to predictively reconstruct, rather than experimentally map a local microcircuit just in terms of the numbers, locations and types of all synapses connecting the neurons. We know that the neuronal branches of neocortical neurons pass close to very many other local neurons. This proximity provides an opportunity for one neuron to form connections with virtually any other without any need to grow towards its target (a tabula rasa principle [60]). We also know that the biological locations of synapses on neuronal arbourizations arise largely by incidental proximity between neighbouring neurons, suggesting that synapse location is largely prescribed by neuronal morphologies (a synapse location principle [61]). The main challenge in predicting the local micro-connectome is therefore to determine which of these close appositions become synapses. When a connection is established between two neocortical neurons, multiple functional synapses are formed, so we know that synapses are formed at several of the prescribed appositions (a multi-synapse principle [62]). We also know that the number of synapses correlates with the number of physical appositions between two neurons, allowing the number of synapses to be predicted by placing model neurons together in three-dimensional space. Being able to predict the number and location of synapses between any two neurons enormously accelerates experimental mapping of all possible connections between any two types of neuron (it takes at least a year to experimentally map the connectivity between two neuron types). Furthermore, the number of boutons an axon can form is also limited and if each connection involves multiple boutons, the average number of neurons it can contact is simply the number of boutons divided by the average number of synapses per connection. Combining these constraints with cell densities makes it possible to predict the probability that any two neurons are synaptically connected [63].

**Figure 2.**
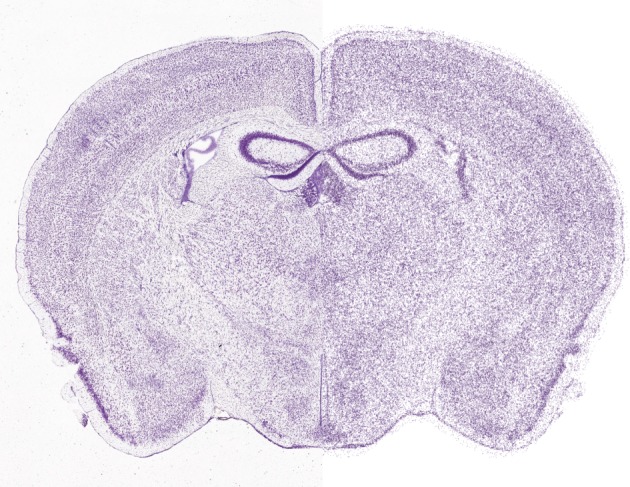
Predictive reconstruction of all cell positions in the whole mouse brain. The left side of the image shows a Nissl-stained section from the Allen Mouse Brain Atlas. The right side shows a digital reconstruction of the cell positions (neurons and glia) obtained algorithmically by analysing the data (C. Erö *et al.* 2014, unpublished data).

**Figure 3.**
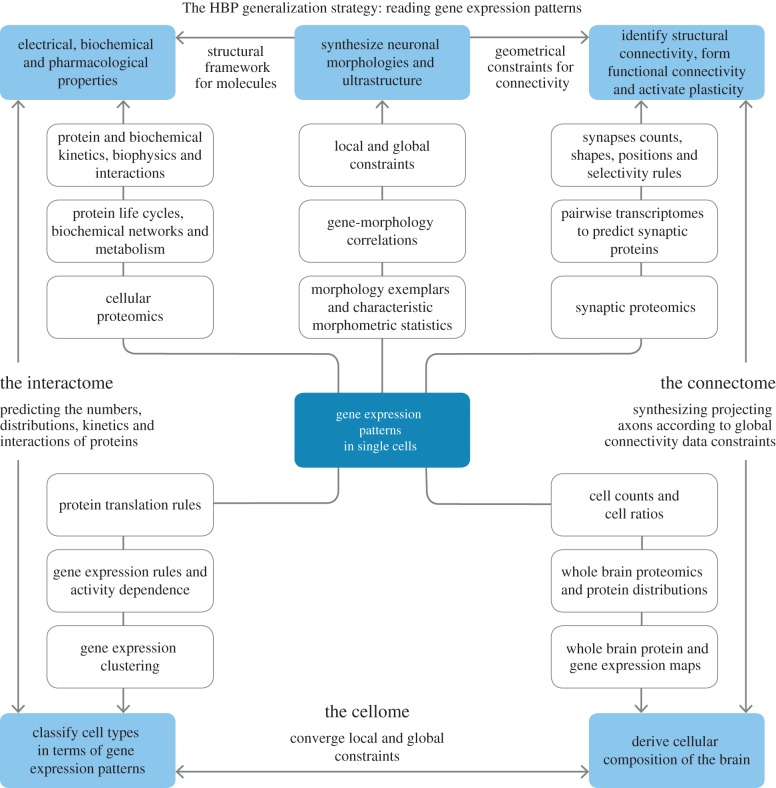
A generic, futuristic schema for predictive reconstruction of a particular instance of a brain (a brain belonging to a specific species with a specific age and gender, and a specific genome). The fundamental data required for future brain mapping come from analysis of gene expression patterns in single cells. These data make it possible to consider clusters of cells with similar patterns as *genetic cell types*. From this starting point, many predictions become possible. By correlating genetic cell types with morphological properties and with constraints on cellular local and global locations (position), it becomes possible to synthesize three-dimensional models of all cell morphologies (including morphologies that have not yet been recorded) and to validate these models against known morphologies. The same data are used to predict cellular composition (at a genetic/molecular level). This is derived by finding the distribution of genetic types that best fits existing whole brain single gene expression maps (in terms of non-negative least squares). The predicted distribution is then validated against whole brain maps of cellular protein expression, thus yielding a complete cellome (the set of all cell types and their respective numbers and distributions). The connectome is derived using brain regional and inter-regional projection data, combined with known principles of synaptic connectivity at the micro-, meso- and macro-levels validated against electron microscopic image stacks from each brain region. Correlations in single-cell transcriptomes of pairs of neurons of known genetic type, and knowledge of the way mRNA is translated into proteins is used to predict the anatomical, physiological, molecular and plasticity properties of the synapses they form and is validated against whole brain maps of synaptic proteins, known synaptic physiology and plasticity. Single-cell gene expression patterns, protein translation principles and protein addressing principles are used to populate and distribute proteins (ion channels, receptors, etc.), peptides, metabolites and other biomolecules within neurons, glia and synapses. Predictions are validated against cellular level gene and protein staining patterns. Reaction kinetics are predicted from fundamental structure–function principles determined in dynamical simulations of molecular interactions. The results can be used to predict a complete interactome and validated against known interactions. At each stage of a reconstruction simulation, results are validated against biophysical, physiological and pharmacological data and where appropriate, against data from cognitive and behavioural studies.

This is just one example of interactions between some important and significant principles (tabula rasa, synapse location and multi-synapse) and parameters (synapses/connection, bouton density, cell density, axonal arbourization and connection probability) of local connectivity that can be exploited to predict a complete set of all possible connections between neurons in a local microcircuit. By analogy, related principles will predictively reconstruct connectivity within and between brain regions to derive an entire connectome. We call this new paradigm of neuroscience, predictive biology. Digital reconstruction provides a way to efficiently apply such principles to data and to test the predictions generated. Figure 2 compares a section of a Nissl-stained whole mouse brain with a digital reconstruction of the cell positions. Figure 3 provides a speculative schema for a future generic approach to predictive reconstruction of the brain, starting at the level of single-cell gene expression data.

Iteration between a predicted reconstruction and new experimental data is integral to refining a reconstruction and improving predictions. Such an iterative process is not possible without big digital science. The strategy of the HBP depends on the realization that a complete dataset from the brain is unattainable. Indeed, there is no such thing as a complete dataset, because, for any given brain, development, time and other contextual factors generate a potential infinity of ‘complete’ sets. What we seek are generalizing principles that make it possible to provide a reconstruction of all potential brains. The solution is to sort out the rules that govern structural and functional principles at each level of organization and between levels. Working in a bottom-up, data-led manner, makes it easier to understand how relatively complex levels of organization are constrained by the principles governing less complex ones. The more precisely such rules are defined, the more constrained the simulation problem will become and the fewer data will be needed. The principle of obtaining more accurate predictions with fewer data is analogous to a similar principle in information theory. We postulate that the main challenge for the next quarter century is not necessarily how to build bigger and bigger datasets. The challenge is to reconstruct a brain from a *minimal dataset*. Even small steps towards complete success will produce deep insights and a parsimonious understanding of the structural and functional organization of the brain. No one knows how many data will be needed in the beginning. To find out, we have to begin the process of simulating and predicting while confronting the predictions with biological reality at each successive stage. The HBP is such a beginning.

It is important to state that this novel approach (data-led simulation modelling) does not constitute a substitute for classical hypothesis-led approaches. Rather, we think of it as a previously untried, complementary methodology that suggests the possibility of developing a general theory of brain organization. By a general theory, we mean a description of the set of rules that determine interactions between biological levels and scales of organization, which ultimately makes it possible to predict how biological variation at one site propagates in terms of its effects to the whole brain, not only spatially, but also in terms of functional integration. Such an achievement would bring major benefits to all of neuroscience, allowing new discoveries to be situated in relation to a concept of the architecture of the brain as a whole. New discoveries would also serve to test the veracity of the described underlying conceptual construct in a recurrent manner.

Other parts of the HBP focus on traditional approaches to neuroscience. In other words, the project should be seen as an organic extension and complement to a tradition that comes before it. For Europe, the HBP is a unique bold step full of scientific risk that offers major potential gains. Brain reconstructions built on fundamental principles and fed pragmatically with available data can help us fill vast gaps in our knowledge and accelerate brain cartography in the next quarter century.

In the last decade, supercomputers have come of age and now have sufficient computing power for the task at hand. Neuroscience could become one of the biggest beneficiaries. With a billion billion calculations per second and multi-scale simulation technology at our disposal, it will become possible to simulate dynamic brain processes at all levels of brain organization, from the microscopic (molecules) to the macroscopic (behaviour). If the project is successful, it will provide opportunities to design revolutionary *in silico* experiments, exploring the mechanisms of cognition, behaviour and disease in ways impossible through laboratory experiments alone. It may also become possible to build a new generation of computers using design principles based on those governing the brain. In that event, neuromorphic computing will begin to complement our present digital computers.

## 7. Medical informatics

Understanding the brain implies that we understand the developmental and ageing trajectory of the brain, the manner in which the natural and social environment shapes age-related changes, and the way in which subtle genetic variations can produce enormous changes in emergent behaviour. It also means understanding similarities and differences in the way the two genders process information, and the locations and ways in which the design principles of the brain break down to cause disease.

Clinical neuroscience is at a similar epistemological crossroads to basic neuroscience. Neurological and psychiatric diseases are both manifestations of abnormal brain function, yet in most countries neurology and psychiatry are separate disciplines. Approaches to treatment are also very different. However, both disciplines have worked and largely still work with symptoms and syndromes, even though in cognitive and behavioural disorders it is often witnesses rather than patients who describe them to physicians. Pathological and normal brain tissue specimens have been historically unattainable in life because the human brain resides in a skull. Post-mortem pathology is of questionable value in identifying disease mechanisms as it represents end-stage disease. It is only since 1973, when the first non-invasive scanning method (X-ray CT) was introduced, that the human brain has begun to give up its secrets in any detail. The power of modern magnetic resonance scanning has been very successful in exploring inter-regional interactions and integration at a mesoscopic scale, for the first time providing detailed physiological and anatomical descriptions of cognitive functions [64]. Today, magnetic resonance promises an ever more sophisticated range of non-invasive methods for characterizing tissue, without the use of ionizing radiation.

Genetic investigation of brain disorders has also yielded fascinating observations. One of the most relevant for our argument is a recent result from a large GWAS, showing that 20 or so haplotypes are strongly associated with psychiatric disease in general, but that none is associated with a specific syndromic category [65,66]. It has been shown, furthermore, that in many disorders haplotypes conferring predisposition to disease contribute little to individual risk. Suggestions that this may be due to cumulative or interacting associations remain to be tested and proved.

In the neurodegenerative diseases, especially those associated with ageing, diagnostic rates are poor. According to recent major pathological reviews, diagnoses of Alzheimer's disease in life are no better than 60–70% accurate [67,68]. Dementia as a syndrome can be caused in a number of ways. The sub-syndromes of dementia do not breed true: a single patient may exhibit evolving features leading to diagnosis of two or three syndromes over a 5–10 year period [69]. Additionally, syndromic diagnoses show little correlation with post-mortem pathology. The mechanisms leading to the various types of dementia remain largely unknown, and it no longer seems as if they are primarily caused by amyloid or by specific disturbances of cholinergic neurons, as was thought until recently. In short, the road from genotype to behavioural phenotype is not a simple one.

The clinical paradigm of functional–structural correlations that started with Broca's observations of language disturbances caused by stroke, and that has so richly served neurologists and psychiatrists over one and a half centuries, has now reached its limits. As the prevalence of some of the least understood diseases of the brain increases, with increasing population age, the search for a new paradigm is vital. The social, economic and emotional ravages that these diseases will produce are inestimable. The brain's large capacity to compensate for neurodegeneration gives grounds for hope. A patient does not present with Parkinson's disease until about 60–70% of a specific bilateral set of nigro-striatal neurons are lost [70]. This provides a window of opportunity for treatment, if we can identify mechanisms suggesting therapeutic targets and achieve diagnosis of pre-clinical disease.

There has been some success in the latter task, especially through the application of machine learning to the analysis of MRI images of the brain [71]. Recent work suggests that identification of pre-clinical pathological states is possible in Huntington's disease [72] and also in Alzheimer's disease.

In brief, informatics and analyses of large datasets offers a possible solution to the impasse in clinical science. The ambition in this area is no smaller than in basic neuroscience, indeed the two areas are closely related. It is clear that a model of the brain could generate hypotheses of disease processes and mechanisms and conversely, that abnormalities associated with disease could serve to reconfigure a normal brain model to assess how they propagate through the various levels of brain organization to their phenotypic manifestations. There is even a theoretical possibility of predicting the effects and side effects of treatments. Congruence between disease manifestations and predictions from a disease-configured brain model would also constitute an excellent test of the veracity of normal brain simulations.

## 8. A strategy for the classification of brain diseases

Neurological and psychiatric diseases are classified in internationally recognized, consensually derived catalogues (ICDM, DSM-IV, DSM-V) that are based on phenotype and backed up by ancillary investigations. Since the introduction of disease genotyping, the emphasis on accurate phenotyping has increased. In our view, this strategy is contentious. Firstly, phenotypes change with time [69]; secondly, they represent interactions with the environment in particular developmental contexts. Indeed, in many ways, it is surprising how useful diagnostic categories have been, especially for diseases whose causes and mechanisms are unknown. As soon as such mechanisms or causes are known, matters change. In infectious diseases of the nervous system, the demonstration of the presence of spirochetal infection at some stage defines syphilis in its myriad manifestations (primary, secondary, tertiary, local, etc.). The same is true for tuberculosis (miliary, meningeal, generalized, pulmonary primary and secondary, etc.). When mechanisms are well understood, for example in brain infections, antibiotic resistance is attenuated by the simultaneous use of multiple drugs that act at different sites in rotation.

There exists a remarkable resource to help combat brain diseases. This is constituted by the enormous amount of data stored in hospital, research and pharmaceutical company databases where it remains, sometimes for a decade or more, often for purely legal reasons. This locked up wealth of information needs to be used rather than ignored, not least because in socialized medical regimes, such as those found in Europe, the taxpayer pays for the infrastructure that collects and stores it. The data should, at least in principle, be made available for studies related to public health as well as for individualized medicine. There are problems, not least those of privacy and informed consent, but there are also technical solutions for dealing with them.

Technical innovation is making it possible to query hospital databases by distributing queries to relevant sites. Databases are left *in situ* in hospitals, where they are protected by the hospitals' own security policies and protocols. Given that original data are never moved, there is no danger that patient records will be corrupted. Files of different types can be accommodated. Search and anonymization can be affected to the highest industry standards, while allowing researchers to receive aggregated results from multiple sites.

Patient records in Europe's hospitals provide a massive amount of data that is potentially analysable by data mining algorithms. Advances in computer science and the widespread application of big data mining have clearly demonstrated that insights can be obtained from noisy, heterogeneous and non-standardized data. Big data analysis lies at the heart of many of our industries and even entertainment. Since its introduction, meteorological forecasts have improved enormously in accuracy. Simulation taking all the details into account has become industry standard practice in aircraft and spacecraft design. Our basic idea is to use data mining to search for *disease signatures*. These will define homogeneous groups of patients characterized by a common set of quantifiable parametrized biological and clinical variables that define the biological make-up of their maladies. The types of data to be incorporated include imaging, electrophysiology, genetics, proteins and other blood variables commonly used in routine clinical practice, results from cerebrospinal fluid investigation and the like. The variety of normal ranges can be dealt with by normalization. ‘Messy’ data can be cleaned up by smoothing—a technique long used in brain imaging to excellent effect. Absent data can be catered for by specialized interpolations, also used effectively in averaging brain images. However, not all issues are resolved. For example, how should one weight the influence of a random blood sugar value to results from genotyping with a million single nuclear polymorphisms (SNPs)?

The underlying strategy is to populate the cerebral disease space with homogeneous groups of patients defined by disease signatures and then to characterise them clinically and biologically. The clinical questions will include—what is the homogeneity of the phenotype associated with a particular *disease signature*? What is the phenotypic difference between close and distant *disease signatures*? Will biological characterisation predict disease manifestations? Does the pattern of biological characteristics suggest a disease mechanism? Are there therapeutic implications? Given that diagnosis by disease signature, based as it is on quantifiable and definable variables, is more accurate than diagnosis based on clinical phenotypes, can we use this information to improve the design of drug trials—using smaller trial populations to achieve adequate power?

**Figure 4.**
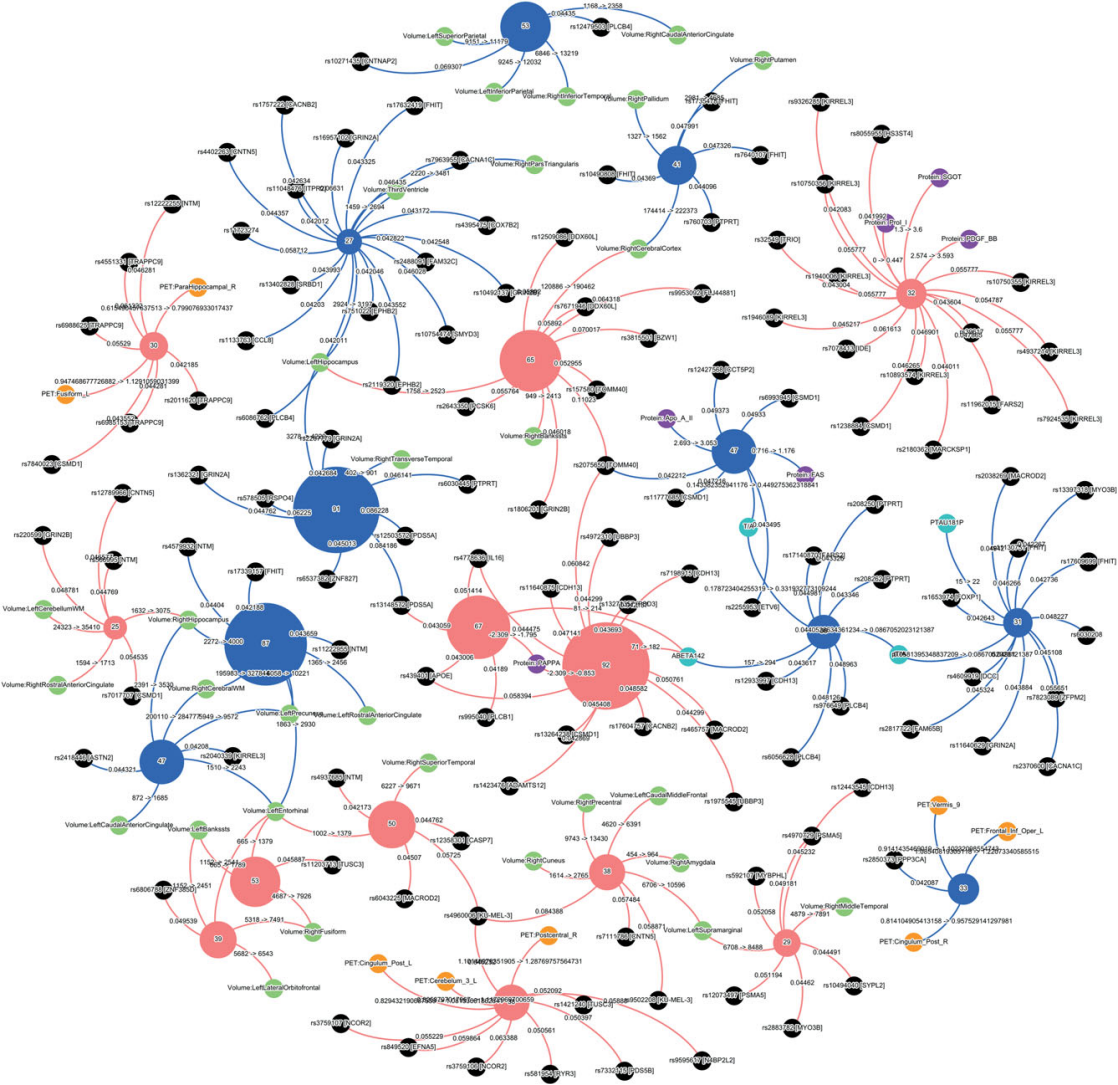
The figure represents subgroups of individuals of advanced age with (red) and without (blue) cognitive symptoms, obtained using an unbiased data clustering method. The datasets contained clinical, imaging, proteomic, cerebrospinal fluid (CSF) protein and genotyping (550–1000 K SNPs) data, some of them incomplete and from three different centres. The method divided the individuals into different subgroups in each category depending on their similarity. The numbers in the subgroups represent the number of individuals belonging to each one. The profile of each subgroup represent specific SNPs (in black), MRI (green) and PET (orange) patterns of focal brain atrophy or hypometabolism, proteins (purple) and CSF proteins (sky blue). The subgroups in the normal cognition category may represent patients with compensated pathology that goes on to cognitive decline as well as completely normal individuals of different biological ages. The cognitive decline category also has a number of subgroups. The largest subgroup (*n* = 92) is associated with Abeta 42 in the CSF and the ApoE4 homozygotic genotype, suggesting it may represent typical Alzheimer disease. The specificity of associations is remarkable. This is an initial, unpublished categorization based on 500 datasets provided by the 3C consortium based in Bordeaux France courtesy of Professors Dartigues and Orgogozo. Although not truly a big data analysis, which would consist of many hundreds of thousands of individuals, the result is striking. (Figure courtesy of HBP sub-project 8, Dr F Kherif and colleagues).

As proof of principle, we have analysed data from 500 people of advanced age and found groupings that associate expected characteristics such as genes, brain atrophy patterns and the like (figure 4). Our study identified a number of dementia types as well as a smaller number of normal groups—as would be expected if some subjects were in pre-clinical compensatory stages of pathology. Although this initial study was based on a small dataset and does not represent a formal proof of concept, the results are very encouraging.

Hospitals, pharmaceutical companies and research groups are providing data for similar studies, including data from the placebo arms of failed trials. Having access to a range of different types of data, from the most structured to the most heterogeneous, will make it possible to investigate issues of stability and sensitivity, required patient numbers and other technical issues. There is still a need to assess data mining algorithms and other engines with the ability to perform complex classification on large and heterogeneous datasets.

The HBP has been running for a year, progress is rapid and will no doubt accelerate in the same way that sequencing the human genome accelerated. We are convinced that if our aims are achieved, we will contribute to a cultural change, characterized by a move away from syndromic diagnosis towards diagnoses based on *biological signatures.* The *signatures* will together constitute a map of brain diseases that will relate to the cartography that represents the biological organization of the brain across scales. In the long run, an individual patient's vector of test results could be compared to such a brain disease classification if it shows sufficient differential sensitivity and accuracy to help diagnosis and precision medicine.

## 9. Adjusting to change

Big science challenges past paradigms and ways of thinking—today no one uses small telescopes for research and no one sequences genes by hand. Few observers continue to question the benefits of big science projects, once they are underway. The momentum and enthusiasm created by new methods have always translated into new jobs, new funding opportunities, new avenues for research and new industries. Disciplines that make effective use of the power of modern computation and data management culture—particle physics, astronomy and genomics—are flourishing.

Without the Human Genome Project, biological and medical research would still be in the dark ages and the lack of clear linear relationships between genes and disease might not have been so obvious. Today, we have hundreds of new companies dedicated to DNA sequencing, gene-based medical diagnostics and related technologies. Furthermore, gene sequencing has become standard practice in a broad range of essential economical activities, including healthcare, drug development, veterinary medicine, agriculture and industrial biotechnology, and has given us the methods we need to monitor and trace potentially pandemic diseases like bird flu and Ebola. According to one study, the Human Genome Project has already yielded a $US 136 return on every dollar of public investment in the United States alone [73]. The European HBP is beginning to have a similar effect. Collaborative brain initiatives are emerging globally with complementary aims. Driven by medical and economic imperatives, many countries are placing brain research on the top of their research agendas and providing new funding for novel approaches. The challenge is enormous and the risks high. However, by not trying, society risks far more. Brain disorders already cost the European economy approximately 800 billion euros a year [74], affecting the lives of some 127 million Europeans. Can we afford not to try new solutions?

Of course, radical change often meets initial opposition. In 1990, for example, *Science* reported an attack on the Human Genome Project, described as ‘mediocre science’ and ‘terrible science policy’ [75]. Critics of ENCODE, an HGP successor project, protested that it was ‘…not the work of scientists' but of a ‘group of badly trained technicians’ [76]. There have been similar polemics around the HBP, published by the journal *Nature* [77]. For neuroscience, the idea of large, non-competitive multi-disciplinary teams of scientists, engineers and clinicians, working to a common vision with a mutually agreed roadmap is a radical one. Using a common ICT infrastructure to reconstruct a synapse, or a neuron, or a whole brain, or to analyse patient data, or to simulate a disease or drug effect, or to build a new computer, or design a robot, are all major intellectual and cultural challenges. To some, the idea of sharing ideas, knowledge, data, and tools before publication are equally frightening. The new way of working challenges existing systems of credits and incentives; new ways of recognizing contributions and promoting careers will be needed. However, other communities such as physics have already faced similar problems. Instead of avoiding the issues, we can use the experience of colleagues in other disciplines to find solutions that work for neuroscience.

Every new technology raises issues of public policy. For instance, the HBP will analyse huge volumes of clinical data from hospital archives, pharmacological company and research databases and other sources, searching for biological signatures of disease. Acquiring these data has already cost trillions of euro of public money, yet the majority remain unused. Exploiting them could bring added value for public health, while simultaneously yielding enormous rewards in terms of better diagnosis, treatment and precision medicine. Technical solutions from modern ICT pave the way for policies that open up clinical data for analysis, while simultaneously providing effective protection for patient privacy.

Maintaining large data resources and new ICT infrastructures will also require new funding models. Which models should be chosen? Maximizing the economic and social benefits of a new model of discovery will require new incentives for sharing data and tools as well as new models making it easier for international teams to share intellectual property. In all these areas, scientists, citizens and patients have the right and duty to challenge decisions by scientists and policy-makers. One of our most important challenges is to find ways for everyone to participate in the debate.

As happens with all disruptive technologies, *big digital science* is rapidly becoming part of our daily lives. We believe that the application of big digital science to neuroscience will provide radically new opportunities to develop a modern cartography of the brain, with a firm grounding in well-established discoveries and methods, allowing us to understand the basic principles of brain architecture and leading us towards theories of the brain, that translate into benefits, not just for medicine but for society.

## 10. Conclusion

The brief we received from the editor of this special issue of *Philosophical Transactions* was to look 25 years into the future of cerebral cartography: to imagine what cerebral cartography will be like then. Already today, cartography has evolved from a way of identifying brain regions and localizing them for use by neurosurgeons, to an anatomical framework on which information about local tissue properties and functions can be distributed to obtain a view of the brain's structural and functional architecture. Informatics and computational power are speeding up this evolution—crossing species boundaries, accounting for inter- and intra-individual variability, providing representations of different levels of brain organization across different spatial and temporal scales. The resulting atlases will become repositories of information associated with particular locations in the brain. Now, we have to use this information to generate a blueprint for brain organization across all scales, to create a theory of brain function, which helps to identify new information needed to progressively and iteratively refine the atlases, and to revise our current classifications of brain diseases, allowing more effective diagnosis, prognosis and treatment. As instigators of the HBP, together with our colleague Prof. Karlheinz Meier, a leader in the field of neuromorphic computing, we have begun to put some of these ideas into practice. We are also content that we seem to have an answer to the philosophical question ‘how can the brain understand itself?’ The answer—‘By exploring itself with the addition of massive computing power and data management capacity, as provided by modern computer science and informatics'.
